# Probiotic Potential of the Marine Isolate *Enterococcus faecium* EA9 and In Vivo Evaluation of Its Antisepsis Action in Rats

**DOI:** 10.3390/md21010045

**Published:** 2023-01-07

**Authors:** Eman H. Zaghloul, Hatem M. Abuohashish, Amany S. El Sharkawy, Eman M. Abbas, Mohammed M. Ahmed, Salim S. Al-Rejaie

**Affiliations:** 1 National Institute of Oceanography and Fisheries (NIOF), Cairo 11516, Egypt; 2 Department of Biomedical Dental Sciences, College of Dentistry, Imam Abdulrahman Bin Faisal University, P.O. Box 1982, Dammam 31441, Saudi Arabia; 3 Department of Pharmacology and Toxicology, College of Pharmacy, King Saud University, Riyadh 11451, Saudi Arabia

**Keywords:** marine, probiotic, *Enterococcus faecium*, sepsis, inflammation, oxidative stress

## Abstract

This study aims to obtain a novel probiotic strain adapted to marine habitats and to assess its antisepsis properties using a cecal ligation and puncture (CLP) model in rodents. The marine *Enterococcus faecium* EA9 was isolated from marine shrimp samples and evaluated for probiotic potential after phenotypical and molecular identification. In septic animals, hepatic and renal tissues were histologically and biochemically evaluated for inflammation and oxidative stress following the probiotic treatment. Moreover, gene expressions of multiple signaling cascades were determined using RT-PCR. EA9 was identified and genotyped as *Enterococcus faecium* with a 99.88% identity. EA9 did not exhibit any signs of hemolysis and survived at low pH and elevated concentrations of bile salts. Moreover, EA9 isolate had antibacterial activity against different pathogenic bacteria and could thrive in 6.5% NaCl. Septic animals treated with EA9 had improved liver and kidney functions, lower inflammatory and lipid peroxidation biomarkers, and enhanced antioxidant enzymes. The CLP-induced necrotic histological changes and altered gene expressions of IL-10, IL-1β, INF-γ, COX-2, SOD-1, SOD-2, HO-1, AKT, mTOR, iNOS, and STAT-3 were abolished by the EA9 probiotic in septic animals. The isolate *Enterococcus faecium* EA9 represents a promising marine probiotic. The in vivo antisepsis testing of EA9 highlighted its potential and effective therapeutic approach.

## 1. Introduction

Lactic acid bacteria (LAB) are widespread microorganisms that exist as normal microflora in humans and animals [1]. Some members of this ecosystem possess probiotic properties that confer a variety of health benefits, including colon cancer prevention, intestinal constipation alleviation [2], serum cholesterol reduction [3], and immunity boosting [4]. Furthermore, they enhance fatty acid absorption in the gut and boost health by producing antioxidants, vitamins, enzymes, organic acids (lactic and acetic), bacteriocins, and other antimicrobial substances [5].

The selection of a probiotic strain is critical since it must be safe with positive impacts on the host. The probiotic strain must be capable of withstanding digestive tract circumstances, such as acidic pH and high concentrations of bile salts and digestive enzymes [6]. Probiotics have been typically studied using culture-dependent methods that rely on isolation, growth, and identification in the lab using standard morphological and biochemical assays. The results of these in vitro tests must be encountered to evaluate and optimize the feeding dose and survivability of the probiotic strain [7] and to determine whether the candidate strain is suitable to be tested in vivo or not. Probiotic strains must be further examined in vivo to assess their ability to control or prevent diseases and disorders [8].

*Enterococci* species are facultative, gram-positive, and anaerobic cocci that can be isolated from multiple environments [9]. The therapeutic utilization of this well-known LAB as a probiotic has been documented in several clinical and preclinical studies [9]. In a recent prospective randomized comparative clinical study, the use of *Enterococcus faecium* isolate as a probiotic showed potential therapeutic effects in 62 patients with post-infectious irritable bowel syndrome [10]. Another *Enterococcus faecium* isolate revealed anti-inflammatory and immunomodulatory effects through the regulation of nuclear factor kappa B (NF-κB) and the c-Jun N-terminal kinase (JNK) pathway [11]. Moreover, the preventive effects of *Enterococcus faecium* probiotic were reported in broiler chickens with necrotic enteritis-induced intestinal barrier injury [12]. *Enterococcus faecium* also potentiated the anti-inflammatory and antiarthritic effects of methotrexate in a rat model of arthritis [13]. In Divyashri et al. study, the probiotic containing *Enterococcus faecium* tended to protect against neurological inflammation and oxidative stress [14].

In the medical field, probiotics have established their effective and potential protective effects against several pathological conditions [15] owing to less frequent antimicrobial resistance in comparison with conventional antimicrobial therapies. The life-threatening septic shock is one pathological condition that was targeted by multiple probiotics due to its high prevalence even with the presence of advanced critical care medical services [16,17]. Clinical data suggested that probiotics might delay the disease onset and reduce inflammatory mediators, particularly in children [18]. Likewise, experimental data demonstrated the ability of probiotics to alleviate sepsis-induced pathological response and inflammatory injuries in several organs [19]. Among the medically introduced probiotics, probiotics isolated from the marine environment have multiple therapeutic applications [20,21]. Their industrial, pharmaceutical, and environmental perspectives were documented [22]. Studies suggested that these marine microorganisms could be a selective source for secondary and bioactive compounds, which could be considered an advantage over the non-aquatic microbes [23]. Accordingly, it could be speculated that the present marine-derived *Enterococcus faecium* EA9 isolate could have potential beneficial properties in sepsis conditions.

Therefore, the current study aimed to isolate and identify a promising LAB from marine samples and assess its probiotic potential. The second aim of the present study was to explore whether the isolated potential probiotic could provide in vivo protective effects in septic Wistar albino rats using the cecal ligation and puncture (CLP) model.

## 2. Results

### 2.1. Assessment of Probiotic Potential of Isolate EA9

#### 2.1.1. Phenotypic and Biochemical Characterization of Isolate EA9

**Figure 1:**
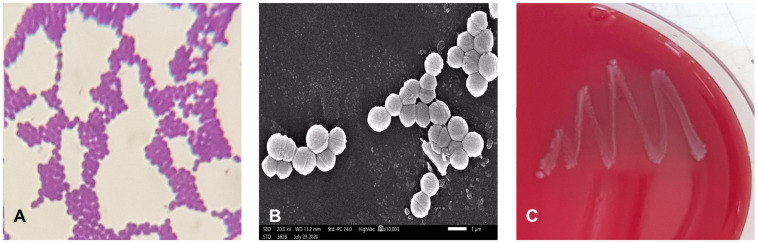
(**A**) Gram staining of isolate EA9 showing the Gram reaction of the isolate. (**B**) EA9 bacterial cells imaged with a scanning electron microscope (SEM) show the EA9 isolate’s unique spherical cell shape and clustering. (**C**) Blood hemolysis test for isolate EA9 after 24 h at 37 °C indicating non-hemolytic activity.

The isolate EA9 was characterized as Gram-positive, catalase negative, non-spore forming, and spherical in shape (Figure 1A,B). The VITEK 2 system was also used to characterize it biochemically, as shown in Table 1. The biochemical features of EA9 VITEK 2 revealed certain biochemical traits, such as lactose fermentation, resistance to four antibiotics (Polymixin, Bacitracin, Novobiocin, and Optochin), and the capacity to survive in 6.5% NaCl. The isolate EA9 was biochemically identified as *Enterococcus faecium* with a 95% likelihood.

#### 2.1.2. Molecular Identification

**Figure 2:**
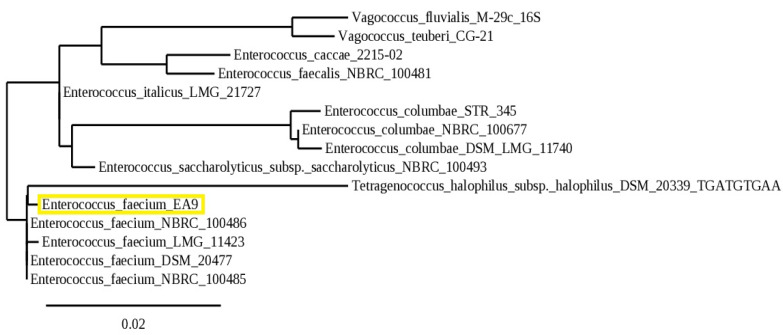
Phylogenetic tree of the marine isolate EA9 (yellow square) based on the 16S rRNA partial gene sequence.

The identity of the marine isolate EA9 was subsequently validated by molecular identification using 16S rRNA gene sequencing analysis and sequence homology searches using the NCBI’s BLAST software (NCBI, Bethesda, MD, USA). With a 99.88% similarity rate, EA9 was identified as *Enterococcus faecium*. The sequence was deposited in GenBank with the accession number MW218438. Figure 2 depicts the evolutionary relationship between the marine isolate EA9 and its close relatives in the NCBI database.

#### 2.1.3. Blood Hemolysis

One of the most important safety factors for evaluating potential probiotic strains is hemolytic activity. EA9 showed no clear or greenish zones around their colonies grown on a blood agar plate, indicating non-hemolytic activity (Figure 1C).

#### 2.1.4. Antibiotic Resistance

The disc diffusion method was used to investigate the antibiotic resistance of the bacterial strain EA9. Out of 11 clinically significant antibiotics examined, isolate EA9 was resistant to 5 types of antibiotics namely: Ampicillin, Ceftriaxone, Piperacillin/Tazobactam, Nalidixic Acid, and Erythromycin (Table 2), whereas it was susceptible to the other 6 antibiotics.

#### 2.1.5. Resistance to pH

**Figure 3:**
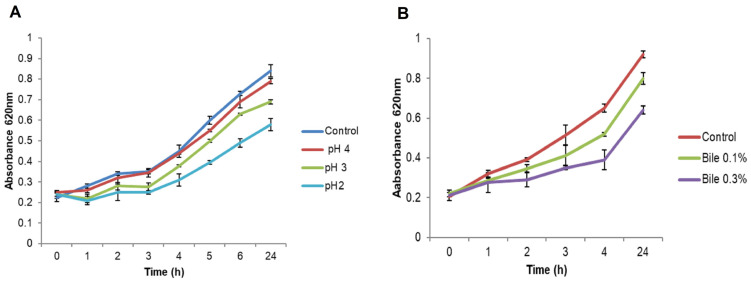
Growth of the marine isolate EA9 at diverse (**A**) pH values and (**B**) concentrations of bile salts for 24 h at 37 °C.

As shown in Figure 3A, the isolate EA9 was able to withstand pH 4 and 3 for 4 h, as shown by an increase in absorbance and survival rates of 98% and 83%, respectively. Similarly, isolate EA9 was able to live and grow at pH 2 for 4 h, although at a slower pace, with a survival rate of 69%, and it continued to develop as reported after 24 h.

#### 2.1.6. Bile Salts Tolerance

Data in Figure 3B shows that the isolate EA9 could withstand 0.1% and 0.3% bile concentrations, as demonstrated by an increase in absorbance and survival rates of 79.7% and 60%, respectively. Furthermore, EA9 continued to develop as observed after 24 h.

#### 2.1.7. Antimicrobial Activity

The antibacterial activity of EA9 cell free supernatant (CFS) was tested against 7 pathogenic bacteria using the agar well-cut diffusion technique. According to the findings, EA9 CFS exhibited potent antimicrobial activity against all pathogens examined, with inhibition zones ranging from 15 to 21 mm in diameter (Table 3). The EA9 CFS showed the highest activity against the Gram-positive bacteria *Staphylococcus aureus* ATCC 25923 (21 mm) while the lowest activity was recorded against the Gram-negative bacteria *Escherichia coli* ATCC 8739 and *Klebsiella pneumonia* ATCC 13883 (15 mm).

### 2.2. In Vivo Testing of the Antisepsis Action of the Isolated EA9 Probiotic

#### 2.2.1. EA9 Effects on Liver and Kidney Functions Tests

Serum levels of liver functions tests, including AST and ALT, as well as the kidney functions tests, including BUN and creatinine, were significantly (*p* ≤ 0.01 and *p* ≤ 0.05; respectively) augmented in CLP groups as compared to the SHAM and SHAM + AE9 groups. Treatment of CLP animals with *Enterococcus faecium* EA9 significantly (*p* ≤ 0.05) lowered the liver and kidney functions tests as compared to the CLP group (Table 4).

#### 2.2.2. EA9 Effects on Liver and Kidney Inflammation

**Figure 4:**
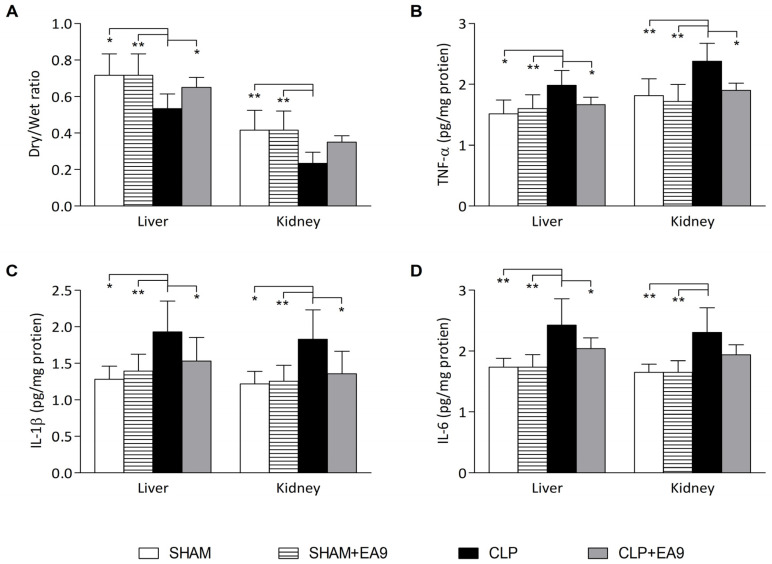
Assessment of inflammation in hepatic and renal tissues following treatment with *Enterococcus faecium* EA9 probiotic in septic Wistar rats. (**A**) degree of edema and fluid infiltration expressed as dry/wet ratio of hepatic and renal tissues, (**B**) hepatic and renal levels of tumor necrosis factor alpha (TNF-α), (**C**) hepatic and renal levels of interleukin-1 beta (IL-1β), and (**D**) hepatic and renal levels of interleukin-6 (IL-6). Data are presented as mean ± SD (*n* = 6) and statistically analyzed by one way ANOVA followed by Tukey-Kramer post hoc test. Significance was considered when * *p* ≤ 0.05 or ** *p* ≤ 0.01.

The dry/wet ratio in the liver samples from CLP group was significantly lower than SHAM (*p* ≤ 0.05), SHAM + EA9 (*p* ≤ 0.01), and CLP + EA9 (*p* ≤ 0.05) groups while the dry/wet ratio in the renal samples from CLP group was significantly lower than SHAM (*p* ≤ 0.01) and SHAM + EA9 (*p* ≤ 0.01) groups (Figure 4A). The levels of inflammatory cytokines, such as TNF-α, IL-1β, and IL-6, were significantly (*p* ≤ 0.05 and *p* ≤ 0.01) higher in liver and kidney samples from CLP group as compared to SHAM and SHAM + EA9 groups. CLP + EA9 showed markedly (*p* ≤ 0.05) lower hepatic levels of TNF-α, IL-1β, and IL-6 as well as renal levels of TNF-α and IL-1β as compared to CLP group (Figure 4B–D).

#### 2.2.3. EA9 Effects on Liver and Kidney Oxidative Stress

**Figure 5:**
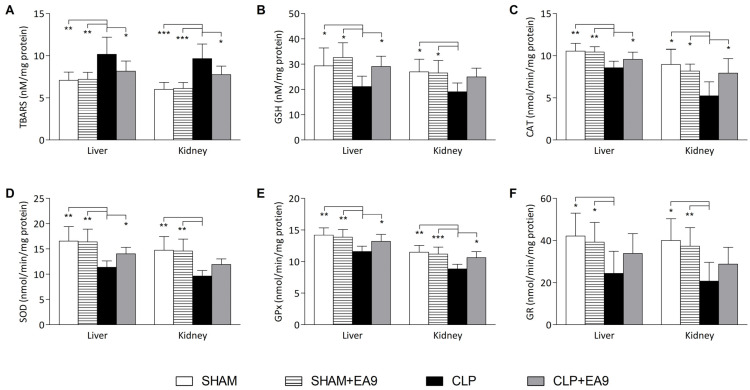
Assessment of oxidative damage in hepatic and renal tissues following treatment with *Enterococcus faecium* EA9 probiotic in septic Wistar rats. (**A**) hepatic and renal levels TBARS as a marker for lipid peroxidation, (**B**) hepatic and renal levels of GSH as an endogenous antioxidant, (**C**) hepatic and renal enzymatic activities of catalase (CAT), (**D**) hepatic and renal enzymatic activities of superoxide dismutase (SOD), (**E**) hepatic and renal enzymatic activities of glutathione peroxidase (GPx), and (**F**) hepatic and renal enzymatic activities of glutathione reeducates (GR). Data are presented as mean ± SD (*n* = 6) and statistically analyzed by one way ANOVA followed by Tukey-Kramer post hoc test. Significance was considered when * *p* ≤ 0.05, ** *p* ≤ 0.01, or *** *p* ≤ 0.001.

In the liver samples, TBARS (*p* ≤ 0.01) level was increased while the levels of GSH (*p* ≤ 0.05) and the enzymatic activities of CAT (*p* ≤ 0.01), SOD (*p* ≤ 0.01), GPx (*p* ≤ 0.01), and GR (*p* ≤ 0.05) were significantly decreased in the CLP group as compared to SHAM and SHAM + EA9 groups. Similarly in the kidney samples, TBARS (*p* ≤ 0.001) was heightened while GSH (*p* ≤ 0.05) levels and the enzymatic activities of CAT (*p* ≤ 0.05), SOD (*p* ≤ 0.01), GPx (*p* ≤ 0.01), and GR (*p* ≤ 0.01) were significantly lowered in the CLP group as compared to SHAM and SHAM + EA9 groups. *Enterococcus faecium* EA9 probiotic treatment to the CLP animals significantly lowered the hepatic and renal TBARS (*p* ≤ 0.05) level and significantly increased the hepatic GSH (*p* ≤ 0.05), hepatic and renal CAT (*p* ≤ 0.05) activity, hepatic SOD (*p* ≤ 0.05) activity, and hepatic and renal GPx (*p* ≤ 0.05) activity (Figure 5A–F).

#### 2.2.4. EA9 Effects on Liver and Kidney Histology

**Figure 6:**
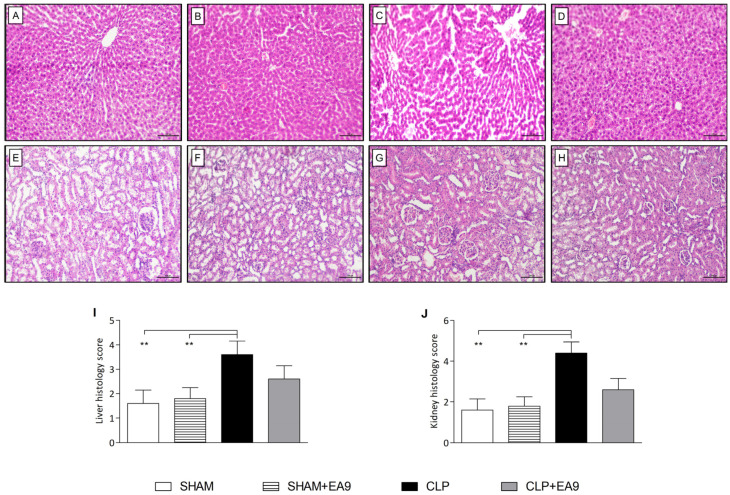
Histological assessment of hepatic (**A**–**D**) and renal (**E**–**H**) tissues following treatment with *Enterococcus faecium* EA9 probiotic in septic Wistar rats (scale bar = 100 µm). (**A**) Liver tissue from sham group showing regular hepatic cells, (**B**) Liver tissue from sham + *Enterococcus faecium* EA9 group showing normal hepatic cells, (**C**) Liver tissue from CLP group showing necrotic hepatic ells with multiple inflammatory infiltration, (**D**) Liver tissue from sham + *Enterococcus faecium* EA9 group showing declined hepatic necrosis and inflammatory cells, (**E**) kidney tissue from sham group showing regular renal structure, (**F**) Kidney tissue from sham + *Enterococcus faecium* EA9 group showing ordinary looking renal tubules, (**G**) Kidney tissue from CLP group showing several necrotic renal ells with diffused inflammatory cells, and (**H**) Kidney tissue from sham + *Enterococcus faecium* EA9 group showing enhanced renal structure and fewer inflammatory cells. Semi-quantitative histological scoring of the (**I**) liver and (**J**) kidney slides. Data are presented as mean ± SD (*n* = 5) and statistically analyzed by Kruskal-Wallis test and Dunn’s multiple comparison post hoc test. Significance was considered when ** *p* ≤ 0.01.

The results of the semiquantitative histological analysis revealed that hepatic and renal slides from SHAM and SHAM + EA9 groups almost normal appearance without inflammatory cells infiltration or necrotic areas. Hepatic and renal slides from CLP group had a significantly (*p* ≤ 0.01) higher number of inflammatory cells infiltration and exudates as well as multiple spots of necrosis as compared to SHAM and SHAM + EA9 groups. Although CLP animals with *Enterococcus faecium* EA9 showed reduced hepatic and renal inflammatory foci and necrotic tissues, the difference from the CLP was not statistically significant (Figure 6A–J).

#### 2.2.5. EA9 Effects on Liver and Kidney Gene Expressions

**Figure 7:**
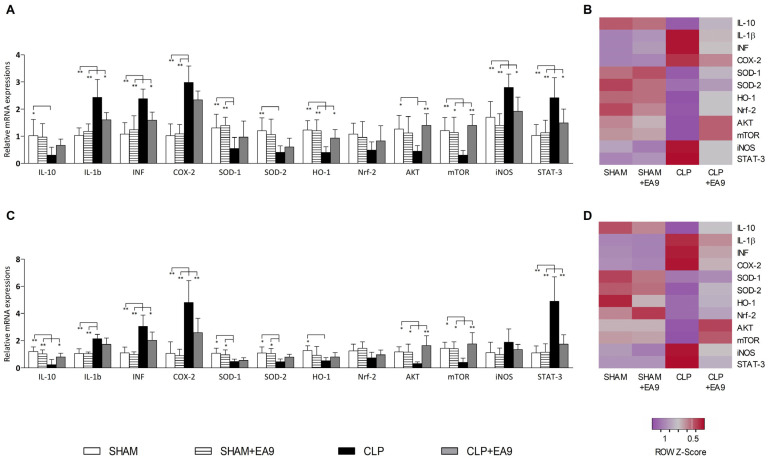
Assessment of mRNA expressions of multiple genes in hepatic (**A**,**B**) and renal (**C**,**D**) tissues following treatment with *Enterococcus faecium* EA9 probiotic in septic Wistar rats. Data are presented as mean ± SD (*n* = 6) and statistically analyzed by one way ANOVA followed by Tukey-Kramer post hoc test. Significance was considered when * *p* ≤ 0.05 or ** *p* ≤ 0.01.

In the hepatic tissues, gene expressions of IL-1β (*p* ≤ 0.01), INF-γ (*p* ≤ 0.01), COX-2 (*p* ≤ 0.01), iNOS (*p* ≤ 0.01), and STAT-3 (*p* ≤ 0.01) were significantly up-regulated while the gene expressions of IL-10 (*p* ≤ 0.05), SOD-1 (*p* ≤ 0.01), SOD-2 (*p* ≤ 0.01), HO-1 (*p* ≤ 0.01), AKT (*p* ≤ 0.05), and mTOR (*p* ≤ 0.01) were significantly down-regulated in the CLP group as compared to SHAM and SHAM + EA9 groups. The treatment of the CLP animals with the probiotic *Enterococcus faecium* EA9 significantly inhibited the hepatic gene expressions of IL-1β (*p* ≤ 0.05), INF-γ (*p* ≤ 0.05), iNOS (*p* ≤ 0.05), and STAT-3 (*p* ≤ 0.05) while it promoted the hepatic gene expressions of HO-1 (*p* ≤ 0.05), AKT (*p* ≤ 0.01), and mTOR (*p* ≤ 0.01) as compared to CLP group (Figure 7A,B). In the renal tissues, gene expressions of IL-1β (*p* ≤ 0.01), INF-γ (*p* ≤ 0.01), COX-2 (*p* ≤ 0.01), and STAT-3 (*p* ≤ 0.01) were significantly up-regulated while the gene expressions of IL-10 (*p* ≤ 0.01), SOD-1 (*p* ≤ 0.05), SOD-2 (*p* ≤ 0.05), HO-1 (*p* ≤ 0.05), AKT (*p* ≤ 0.05), and mTOR (*p* ≤ 0.05) were significantly down-regulated in the CLP group as compared to SHAM and SHAM + EA9 groups. Septic animals with the *Enterococcus faecium* EA9 treatment has significantly lower renal gene expressions of INF-γ (*p* ≤ 0.05), COX-2 (*p* ≤ 0.01), and STAT-3 (*p* ≤ 0.15), along with enhanced renal gene expressions of IL-10 (*p* ≤ 0.05), AKT (*p* ≤ 0.01), and mTOR (*p* ≤ 0.01) as compared to the CLP group (Figure 7C,D).

## 3. Discussion

The marine environment represents an untapped niche for novel LAB isolates [24]. Marine LAB have promising possibilities as probiotics and can be considered as a source of new bioactive compounds since their characteristics and biological activity vary from their terrestrial counterparts. Their ability to live under the harsh marine circumstances makes them more competitive than their terrestrial counterparts. Therefore, the marine isolate *Enterococcus faecium* EA9 was assessed in this study for the primary safety criteria for use as a probiotic. Additionally, the antisepsis effects of *Enterococcus faecium* EA9 probiotic was evaluated as a potential therapeutic application using an in vivo model of sepsis in rats.

*Enterococcus faecium* EA9 demonstrated non-hemolytic activity, which confirms its safety. Previous studies have also indicated the non-hemolytic ability of *Enterococcus* sp. Pieniz et al. [25] observed that *Enterococcus durans* strain LAB18s isolated from Minas Frescal cheese did not possess any hemolytic activity. Moreover, De Vuyst et al. [26] found that several *Enterococcus* species collected from various environments were all non-hemolytic. The results showed that the marine isolate *Enterococcus faecium* EA9 is sensitive to 6 types of the tested antibiotics. On the other hand, EA9 showed resistance to 5 types of antibiotics, which is a very important criterion in developing combination therapies of antibiotic/probiotic [27]. The probiotic strain must be able to survive in presence of some types of antibiotics. These antibiotics are widely employed in medication and as preservatives in food products; as a result, a probiotic strain that is highly susceptible to most antibiotics will be quickly cleared from the gastrointestinal system, rendering it useless [28]. The antibiotic resistance genes of many probiotic strains are found in a non-transferable chromosome [29], which reduce the likelihood of transmission to other bacteria in the gut [30]. In addition, EA9 was shown to be sensitive to vancomycin, which is noteworthy given that vancomycin is often employed as a last-resort antibiotic owing to its efficiency against multiple drug-resistant bacteria. Furthermore, the resistance of probiotics to various antibiotics has previously been reported by Bs et al. [28], who reported that *Enterococcus faecium* BS5 is susceptible to tetracycline, rifamycin, and chloramphenicol but resistant to erythromycin, kanamycin, penicillin, streptomycin, and ampicillin. Notably, different probiotic strains often exhibit varying degrees of resistance to various kinds of antibiotics [31]. For instance, *Lactobacillus bulgaricus* and *Streptococcus thermophilus* have been shown to have a high level of resistance to kanamycin, ampicillin, and streptomycin [32].

*Enterococcus faecium* EA9 showed a good potential to resist hard surviving conditions when the pH values were reduced to 4, 3, and 2. The survival rates of *Enterococcus faecium* EA9 in this condition was 98%, 84%, and 69%. Similarly, Pimentel et al. [33] obtained 73 *enterococci* isolate from Terrincho cheese, and he reported that most of the obtained *Enterococcus durans* and *Enterococcus faecium* strains were resistant to a low pH environment. Bs et al. [28] recorded the strong ability of *Enterococcus faecium* BS5 to withstand low pH with respective survival rates of 78% (pH 4), 78% (pH 3), and 25% (pH 2). Moreover, *Enterococcus faecium* EA9 managed to endure high concentrations of bile salts, which is compatible with Bs et al. [28], who discovered that *Enterococcus faecium* BS5 managed to endure and grow at varied bile contents. Since bile salts alter the cell membrane structure, they are very hazardous to living organisms. The probiotic strains’ resilience to high bile contents is mainly due to bile salts hydrolase (BSH), which hydrolyzes the toxic conjugated bile salts [34]. The capacity of the probiotic strain to tolerate the circumstances of the small intestine could be more crucial than the capacity to withstand low pH. Some investigations suggest that acid labile bacteria may be buffered throughout the stomach. The probiotic strain should, however, be able to endure destruction by bile salts and hydrolytic enzymes present in the intestine and must be able to survive and colonize there in order to be beneficial to the host. This trait is crucial for the choice of innovative probiotic strains [25].

*Enterococcus faecium* EA9 was reported to elicit potential antibacterial efficacy against all of the investigated pathogenic strains. This is critical when choosing probiotic strains since they must be capable of eliminating competitors. LAB generates a variety of antimicrobial agents, giving it an edge over other gut pathogens [35]. The production of the antimicrobial agents is highly affected by strain and growth condition differences [36]. Among the many antimicrobial agents produced by LAB are lactic acid, fatty acids, acetic acid, inhibitory peptides (bacteriocins), and hydrogen peroxide. *Enterococci* produce bacteriocins called enterocins, which have been isolated and described in many *enterococci* [37].

In the second part of the study, the antisepsis effects of the marine isolate *Enterococcus faecium* EA9 were assessed in a CLP model, which creates an intensive pathological environment characterized by cellular inflammation and oxidative stress in multiple organs including the liver [38] and kidneys [39]. The CLP resulted in severe alterations in the histological structure of hepatic and renal cells along with impaired hepatic and renal functions. Additionally, the reduced dry/wet ratio in the CLP group indicated the development of inflammatory edema. The marked increase in TNF-α, IL-1β, and IL-6 also indicated the inflammatory response. Studies proposed oxidative stress and CLP linkage, where markers of oxidative stress are triggered by CLP along with suppressed endogenous antioxidant protective mechanisms [40,41]. Likewise, our biochemical findings revealed the augmentation of cellular membrane lipid peroxidation markers (TBARS) as well as reduced endogenous sulfhydryl containing-antioxidant (GSH) and suppressed antioxidant enzymes in the CLP group, which indicates the extensive production of free radicals and decreased antioxidant defensive capabilities. This data implies that the CLP model was a successful model to evaluate the antisepsis potential of the isolate *Enterococcus faecium* EA9 probiotic.

The marine origin of the isolate *Enterococcus faecium* EA9 probiotic supported the fact that several marine-derived microorganisms and the associated-bioactive compounds could have possible medical applications, such as regulating blood pressure [42], hepatoprotective effects and antioxidant potentials [43], suppression of intestinal inflammation [44], antitumor activity [45,46], and antibacterial activity [47]. In the present study, the marine isolate *Enterococcus faecium* EA9 probiotic markedly reduced the pathological consequences of sepsis in both hepatic and renal tissues. This was indicated by the significant improvement of hepatic and renal function tests along with less reported necrotic and inflammatory spots in liver and kidney samples. Furthermore, the inflammatory parameters including dry/wet ratio and inflammatory cytokines were suppressed by the assessed probiotic. These cytokines are secreted by immune cells in response to sepsis to promote inflammation as a kind of defense mechanism to protect cellular integrity and maintain cellular functions [48]. This will create a harmful environment of free radicals. Therefore, the oxidative stress and lipid peroxidation biomarkers were augmented in septic animals. The free radicals alleviating effects of EA9 probiotic markedly suppressed lipid peroxidation and enhanced the antioxidant mechanisms, which prevented further cellular damage. Interestingly, antioxidant genes, such as CAT, SOD, and GR, are expressed, identified, and characterized in marine microorganisms [49,50,51].

Gene expression of IL-10, IL-1β, INF-γ, and COX-2 was determined as an indicator for inflammatory response. IL-10 is an anti-inflammatory cytokine while IL-1β, INF-γ, and COX-2 are expressed during pathological inflammatory conditions, such as sepsis [52,53,54]. The anti-inflammatory effects of *Enterococcus faecium* EA9 probiotic were obvious at the gene level, where hepatic tissues had significantly reduced IL-1β and INF-γ gene expressions while renal tissues had significantly improved IL-10 and reduced INF-γ and COX-2 gene expressions. The SOD-1 gene encodes for the cytoplasmic SOD while SOD-2 is present in the mitochondria. Both enzymes catalyze the transformation of harmful free radicals to less reactive oxygen species. Although *Enterococcus faecium* EA9 probiotic enhanced the CLP-suppressed SOD activity in the hepatic tissues, its effect was not prominent on the gene level. HO-1 is a critical cytoprotective factor that maintains antioxidant/oxidant homeostasis in case of cellular stress and inflammation [55]. HO-1 and Nrf-2 are regulator for cellular oxidant resistance [56]. *Enterococcus faecium* EA9 probiotic abolished sepsis-induced down-regulation of HO-1 in the liver. Moreover, we did not report a significant effect of CLP or *Enterococcus faecium* EA9 probiotic on Nrf-2 gene expression. AKT/mTOR signaling pathway is well-known as a regulator of cell cycle and apoptosis. The sepsis protective effects following activation of this pathway have been documented in previous studies [57,58]. Our results showed similar effects, where *Enterococcus faecium* EA9 probiotic improved gene expression of AKT in both tissues and mTOR in kidney samples. The iNOS stimulation during sepsis might result in elevated levels of NO, which is associated with the development of nitrosative stress and reactive nitrogen species [59]. *Enterococcus faecium* EA9 probiotic inhibited iNOS gene expression in the liver tissues suggesting a site-specific effect of the probiotic in relation to the iNOS pathway. STAT-3 is a transcription factor that regulates multiple inflammatory cascades in many infectious and autoimmunity conditions, such as sepsis [60]. In line with the biochemical, histological, and molecular analysis of the inflammatory response, the STAT-3 gene was markedly up-regulated in the CLP group while *Enterococcus faecium* EA9 probiotic treatment lowered its expression in hepatic and renal tissues.

Despite our efforts to reduce variabilities during the surgical procedure by controlling multiple factors, such as differences in the ligated length of the cecum, the size and number of punctures, and the amount the extruded stool, the procedure remains subjective. Furthermore, some revealing factors of the antisepsis effects of the tested probiotic were not recorded, such as body temperature and survival rate, which could have added further evidence of the protective antisepsis effects of the probiotic.

## 4. Materials and Methods

### 4.1. Assessment of Probiotic Potential of Isolate EA9

#### 4.1.1. Phenotypic and Biochemical Characterization of Isolate EA9

For the biochemical identification and characterization of isolate EA9, overnight culture of EA9 were inoculated in Gram-positive (GP) cards and run on the VITEK 2 compact fully automated microbial identification system version 07.01 (BioMérieux, Craponne, France). The system analyzes the cells for sugar fermentation, antibiotic resistance, and enzyme hydrolysis, among other biochemical assays. Moreover, scanning electron microscope (SEM) (JSM-5300, JEOL, Tokyo, Japan) was also used to observe the typical cell shape and size of isolate EA9 [61].

#### 4.1.2. Molecular Identification and Phylogenetic Analysis of Isolate EA9

*Enterococcus faecium* EA9 was grown for 24 h at 37 °C in MRS medium, then the total genomic DNA of EA9 was extracted and purified according to the manufacturer’s instructions for the DNA extraction kit (QIAGEN, Hilden, Germany). The prepared DNA was loaded with the loading dye in a 1% agarose gel prepared in Tris EDTA Acetic acid (TEA) buffer with 1 μg/mL ethidium bromide (Sigma, St. Louis, MO, USA), and the voltage was applied (90 *v*/cm) for electrophoresis after soaking in TEA buffer. The UV transilluminator (Bio-Rad, Hercules, CA, USA) was used to visualize the bands that had been obtained. For molecular identification of the marine isolate EA9, the PCR product for EA9 16S rRNA gene was performed and sequenced by Applied Biotechnology Co., Ismailia, Egypt. Under the accession number MW218438, the obtained sequence was submitted to the National Center for Biotechnology Information (NCBI) GenBank. Furthermore, the sequence was examined using NCBI’s BLASTn tools, which revealed that isolate EA9 has a 99.88% similarity with *Enterococcus faecium* strain DSM 20477 (accession number NR114742.1). The isolate EA9 16S rRNA gene sequence was then aligned with related species using the ClustalW tool (Version 2.1), and a phylogenetic tree was generated through www.phylogeny.fr (accessed on 12 February 2021) using the Maximum Likelihood technique [62].

#### 4.1.3. Hemolytic Activity

Blood agar plates (supplemented with 7% human blood) were streaked with isolate EA9 and incubated at 37 °C for 24 h to test hemolytic activity. The plates were then examined for evidence of blood hemolysis, such as the creation of clear zones (β-hemolysis), development of green zones of partial hemolysis (α-hemolysis), or the absence of clear zones around the colonies (γ-hemolysis) [63].

#### 4.1.4. Antibiotic Susceptibility

The antibiotic susceptibility of EA9 was determined using the Kirby-Bauer disc diffusion method. Antibiotic discs (Oxoid, Basingstoke, UK) of various types were placed on the surface of agar plates that had previously been inoculated with EA9. After a 24 h incubation period at 37 °C, the plates were checked for the development of clear zones around discs, indicating EA9 sensitivity to the tested antibiotic [64].

#### 4.1.5. Resistance to Low pH

The ability of isolate EA9 to withstand low pH levels was evaluated according to Nawaz et al. study [65]. The isolate was inoculated (1% *v*/*v*) into sterile MRS broth adjusted to several pH values (2, 3, 4, and 6.5) with 0.1 N HCl and incubated at 37 °C. The absorbance was measured at 620 nm using a spectrophotometer (Unico, Franksville, WI, USA) at hourly intervals for 6 h. The isolate EA9 was cultured at various pH levels to assess its capacity to resist low pH conditions, mimicking the pH of the stomach, which is estimated to be around 3 with a stay period of about 4 h.

#### 4.1.6. Bile Salts Tolerance

The ability of isolate EA9 to withstand high bile salts concentrations was investigated according to the method described by Harsa and Yavuzdurmaz [66]. An overnight culture of EA9 (1% *v*/*v*) was inoculated in 15 mL sterile MRS broth with various bile salts concentrations (0, 0.1, and 0.3% (*w*/*v*)) and incubated for 4 h at 37 °C. The absorbance of the culture was measured at hourly intervals with a spectrophotometer (Unico, Franksville, WI, USA) at 620 nm. The average bile salts content in the intestine is variable, although it is approximated to be around 0.3% *w*/*v*, with a stay time of 4 h.

#### 4.1.7. Antimicrobial Activity

Different Gram-negative bacterial pathogens (*Klebsiella pneumonia* ATCC 13883, *Escherichia coli* ATCC 8739, *Aeromonas hydrophila* ATCC 13037, *Pseudomonas fleurescence* ATCC 13525) as well as Gram-positive bacterial pathogens (*Staphylococcus aureus* ATCC 25923, *Enterococcus faecalis* ATCC 29212, *Streptococcus agalactiae* ATCC 13813) were used to evaluate the antimicrobial activity of isolate EA9 using the agar well cut diffusion technique. Each pathogenic strain was inoculated (1% *v*/*v*) in nutrient agar plates. Using a sterile cork borer, wells of 8 mm diameter were cut in solidified agar and filled with 100 μL of filter sterilized cell free supernatant (CFS) of EA9. The antibacterial activity was assessed by measuring the clear zone diameter around each well after 24 h of incubation at 37 °C.

### 4.2. In Vivo Testing of the Antisepsis Action of the Isolated EA9 Probiotic

#### 4.2.1. Ethical Approval and Experimental Model

The experimental animal part of this study was conducted in the animal house at Institute of Graduate Studies and Research, Alexandria University under an ethical approval number AU14-210126-3-2. The experimental procedures were in accordance with the 8th edition of the National Institute of Health (NIH) guidelines for the care and use of laboratory animals and ARRIVE guidelines. A number of twenty-four healthy male Wistar albino rats weighting approximately 200 g were included in this study, whereas animals with abnormalities or low weight were excluded. Animals were simply randomized into four groups (*n* = 6) in labeled polystyrene cages. The first group (SHAM) included animals, who were subjected to the surgical procedure without the ligation and puncture steps. The second group (SHAM + EA9) included animals, who were subjected to the surgical procedure without the ligation and puncture steps as well as probiotic pretreatment for 10 days before the surgical procedure. The third group (CLP) included animals, who were subjected to the CLP procedure. The fourth group (CLP + EA9) included animals, who were subjected to the CLP procedure as well as probiotic pretreatment for 10 days before the surgical procedure. The sepsis model (CLP surgical procedure) was preformed according to the method described by Zubrow et al. [67]. The cecum of the generally anesthetized animals by 60 mg/kg ketamine and 5 mg/kg xylazine was visualized and exposed after a median laparotomy incision of 15 mm long. Then, the distal portion of the cecum was ligated with a sterile 4-0 silk thread. After this, the ligated sections were exposed to 2 or 3 punctures using an 18-gauge syringe needle followed by squeezing the small volume of the feces. A sterile saline (20 mL/kg body weight) were provided subcutaneously, and the ligated tissues were replaced in the abdominal cavity. Pain following the surgical procedures was controlled by ketorolac (30 mg/kg) every 12 h, intraperitoneally. Ten days before the sepsis model, the tested EA9 probiotic in a freeze-dried format was diluted in 1 M of NaCl solution (1 × 10^7^ CFU/mL) and a 250 μL (per animal) were provided consecutively and blindly by oral gavage to animals in the third and fourth groups. Three days after the CLP, all rats were euthanized with a lethal inhaled dose of general anesthesia, and the biological samples including blood, liver, and kidneys were collected for histological, biochemical, and molecular analysis.

#### 4.2.2. Serum Biochemistry

Blood samples were centrifugated at 4000 RPM for 15 min to obtain serum samples, which were used to determine the concentrations of serum aspartate aminotransferase (AST) and serum alanine transaminase (ALT) as markers for liver disfunction, as well as blood urea nitrogen (BUN) and creatinine as markers for renal disfunction using a fully-automated Cobas C311 chemistry analyzer (Roche diagnostics Ltd., Risch-Rotkreuz, Switzerland).

#### 4.2.3. Evaluation of Inflammation

The concentrations of inflammatory cytokines, such as tumor necrosis factor alpha (TNF-α), interleukin-6 (IL-6), and interleukin-1 beta (IL-1β) in the hepatic and renal homogenates, were measured with an enzyme-linked immunosorbent assay (ELISA) technique following instructions provided by the kits (R & D systems Inc., Minneapolis, MN, USA). Tissue edemas were determined by measuring wet/dry. In brief, a small portion from liver and kidney samples were isolated and weighed (wet weight). These portions were re-weighed after drying (dry weight) overnight at 60 °C.

#### 4.2.4. Evaluation of Oxidative Damage

After homogenization in a phosphate buffer (1:10, *w*/*v*), the liver and kidney homogenates were used to determine the levels of thiobarbituric acid reactive substances (TBARS), as a lipid peroxidation biomarker and reduced glutathione (GSH), and as an endogenous antioxidant, using commercial diagnostic kits (Cayman Chemical Co., Ann Arbor, MI, USA). Though, the post-mitochondria supernatants obtained after centrifugation of the homogenates were used to determine the enzymatic activities of catalase (CAT), superoxide dismutase (SOD), glutathione reeducates (GR), and glutathione peroxidase (GPx) using their commercially assay kits (R & D systems Inc., Minneapolis, MN, USA).

#### 4.2.5. Histological Evaluation

Liver and kidney samples were collected in 10% formalin solution, fixed in plastic cassettes, and dehydrated by graded concentration of ethanol. Afterward, each sample was embedded in a single paraffin block. All blocks were sectioned using a microtome into a 4 µm thickness sections. After picking up on glass slides, these sections were stained with hematoxylin and eosin (H & E) for histological evaluation. The stained slides were assessed microscopically and scored in a blind manner. Tissue necrosis was evaluated based on the following criteria: (score-0) normal looking cells with no necrosis, (score-1) 1 or 2 spots of necrotic tissues, (score-2) 3 or 5 spots necrotic tissues, and (score-3) more than 5 spots necrotic tissues. Tissue inflammation was assessed according to the following measures: (score-0) normal looking cells lacking inflammatory cells, (score-1) scattered inflammatory cells, (score-2) inflammatory foci, and (score-3) multiple diffused inflammatory cells. The final necrotic and inflammatory scores were summed and presented per each group.

#### 4.2.6. Polymerase Chain Reaction (PCR) Assessment of Genes Expressions

Total RNA was extracted from the liver and kidney samples using the TRIzol method (easy-RED, iNtRON Biotechnology). At the end of the experiment, RNA purity was determined at 260/280 nm by a NanoDrop system (BioDrop, Biochrom Ltd., Cambridge, UK). The cDNA (1 ng/uL) was synthesized from the RNA samples that showed the highest A260/A280 ratio by adding DNase I (New England Biolabs, Ipswich, MA, USA) as the template. The reverse transcriptase (RT-PCR beads, Enzynomics, Daejeon, Korea) was employed, and the PCR amplification was performed by (Applied Biosystems, Veriti 96-Well Thermal Cycler, Waltham, MA, USA). The synthetized cDNA was incorporated in the Real-Time PCR reaction (Bico, Thermo-Fisher, Waltham, MA, USA) under the following procedure: 15 min of initial denaturation (95 °C) followed by 40 cycles of 95 °C for 10 s, 55–60 °C for 20 s, and 72 °C for 30 s. Unique and specific products were noticed when a melting curve at the end of the last cycle where the temperature increased from (55–60 to 95 °C) in increments of 0.5 °C. Primers list of the targeted genes in this study are present in Table 5. β-actin served as a housekeeping gene. The values given out in an n-fold difference relative to the calibrator (control) when the 2^ΔΔCt^ method was applied to normalize the critical threshold (Ct) quantities of target genes relative to those of β-actin [68].

#### 4.2.7. Statistical Analysis

The normality of the data was assessed with the Shapiro-Wilk test. Results of the biochemical and molecular analysis were expressed as mean ± standard deviation (SD) and statistically analyzed using one way analysis of variance (ANOVA) followed by of Tukey-Kramer post hoc test. Scores of the histological evaluation of necrosis and inflammation were also expressed as mean ± SD and analyzed by Kruskal-Wallis test and Dunn’s multiple comparison post hoc test. Means differences were judged as significant when *p* values were ≤ 0.05. GraphPad Prism 5 (GraphPad Software, Inc., La Jolla, CA, USA) was utilized as the analysis software. Heatmaps were executed using Heatmapper (Wishart Research Group, University of Alberta, Ottawa, Canada) [69]. Graphical abstract was prepared using Biorender.com (Agreement number: FV24JOFCRX, 20 October 2022).

## 5. Conclusions

In the present work, the marine isolate EA9 was tested for its safety and probiotic potential in addition to its possible health benefits. According to the acquired results, this isolate was identified as *Enterococcus faecium*, and it possesses potential probiotic and technical qualities, include resistance to low pH, high bile salts concentrations, and broad antibacterial activity. In addition, the results of the in vivo investigation show that *Enterococcus faecium* EA9 isolate may have possible therapeutic use in pathological conditions, where inflammation and oxidative stress are deemed as contributing factors. The efficacy against experimental sepsis was proven in this study while future research might provide further evidence for other medical applications of the probiotic.

## Figures and Tables

**Table 1:** Biochemical characterization of the marine isolate EA9 using the VITEK 2 microbial identification system (BioMérieux, Craponne, France).

Test	Result	Test	Result
Alpha-mannosidase (AMAN)	*−*	d-mannosE (dMNE)	+
Phosphatase (PHOS)	−	Methyl-b-d-glucopyranoside (MBdG)	+
Leucine arylamidase (LeuA)	−	Pullulan (PUL)	−
l-proline arylamidase (ProA)	−	d-raffinose (dRAF)	−
Tyrosine arylamidase (TyrA)	−	Optochin resistance (OPTO)	+
d-sorbitol (dSOR)	−	Urease (URE)	−
Polymixin b resistance (POLYB)	+	d-mannitol (dMAN)	−
d-amygdalin (AMY)	−	d-galactose (dGAL)	+
Phosphatidylinositol phospholipase (PIPLC)	−	d-ribose (dRIB)	+
d-xylose (dXYL)	−	l-lactate alkalinization (ILATK)	−
Arginine dihydrolase 1 (ADH1)	+	Lactose (LAC)	+
Beta-galactosidase (BGAL)	+	N-acetyl-d-glucosamine (NAG)	+
Alpha-glucosidase (AGLU)	−	d-maltose (dMAL)	+
Beta glucuronidase (BGURr)	−	O/129 resistance (comp. vibrio.) (O129R)	+
Alpha-galactosidase (AGAL)	−	Salicin (SAL)	+
l-pyrrolydonyl-arylamidase (PyrA)	+	Saccharose/Sucrose (SAC)	−
Beta-glucuronidase (BGUR)	−	d-trehalose (dTRE)	+
Alanine arylamidase (AlaA)	−	Arginine dihydrolase 2 (ADH2s)	+
Ala-phe-pro arylamidase (APPA)	−	Bacitracin resistance (BACI)	+
Cyclodextrin (CDEX)	+	Novobiocin resistance (NOVO)	+
l-aspartate arylamidase (AspA)	−	Growth in 6.5% NaCl (NC6.5)	+
Beta galactopyranosidase (BGAR)	−		

“+” indicates positive test results and “−” indicates negative test results according to the manufacturer.

**Table 2:** Antibiotic resistance of isolate EA9.

Antibiotic	Result
Nalidixic Acid (30 mcg)	R
Ampicillin (10 mcg)	R
Erythromycin (15 mcg)	R
Tetracycline (30 mcg)	S
Piperacillin/Tazobactam (110 mcg)	R
Vancomycin (30 mcg)	S
Oxacillin (1 mcg)	S
Ceftriaxone (30 mcg)	R
Amoxicillin (25 mcg)	S
Ofloxacin (5 mcg)	S
Cephradine (5 mcg)	S

“R” resistant and “S” sensitive.

**Table 3:** Antimicrobial activity of isolate EA9 cell free supernatant against different indicator pathogens.

Indicator Pathogen	Inhibition Zone Diameter (mm)
*Pseudomonas fluorescens* ATCC 13525	18 ± 0.5
*Streptococcus agalactiae* ATCC 13813	19 ± 0.44
*Aeromonas hydrophila* ATCC 13037	18 ± 0.23
*Staphylococcus aureus* ATCC 25923	21 ± 0.115
*Escherichia coli* ATCC 8739	15 ± 0.05
*Enterococcus faecalis* ATCC 29212	18 ± 0.15
*Klebsiella pneumonia* ATCC 13883	15 ± 0.23

**Table 4:** Assessment of serum biochemistry following treatment with *Enterococcus faecium* EA9 probiotic in septic Wistar rats.

	SHAM	SHAM + EA9	CLP	CLP + EA9
**AST**	143.83 ± 13.79 **	141.16 ± 20.06 **	211 ± 25.49	164.5 ± 41.92 *
**ALT**	57.33 ± 8.94 **	55.33 ± 7.89 **	83.5 ± 13.14	63 ± 12.17 *
**BUN**	72.16 ± 14.77 *	72.51 ± 16.00 *	104 ± 28.09	74.66 ± 7.89 *
**Creatinine**	0.71 ± 0.09 *	0.71 ± 0.08 *	1.1 ± 0.33	0.73 ± 0.15 *

Data are presented as mean ± SD (*n* = 6) and statistically analyzed by one way ANOVA followed by Tukey-Kramer post hoc test. Significance was considered when * *p* ≤ 0.05 or ** *p* ≤ 0.01 vs. CLP.

**Table 5:** Primers sequences according to NCBI gene database.

Gene Name	Accession Number	Sequences
Interleukin-10 (IL-10)	XM_006249712.4	F: 5′-TGCCTTCAGTCAAGTGAAGAC-3′
		R: 5′-AAACTCATTCATGGCCTTGTA-3′
Interleukin-1β (IL-1β)	NM_031512.2	F: 5′-CACCTCTCAAGCAGAGCACAG-3′
		R: 5′-GGGTTCCATGGTGAAGTCAAC-3′
Interferon-γ (INF-γ)	NM_138880.3	F: 5′-GTGTCATCGAATCGCACCTG-3′
		R: 5′-GTTCACCTCGAACTTGGCGA-3′
Cyclooxygenase-2 (COX-2)	S67722.1	F: 5′-TGAGTACCGCAAACGCTTCT-3′
		R: 5′-ACACAGGAATCTTCACAAATGGA-3′
Superoxide dismutase-1 (SOD-1)	NM_017050.1.1	F: 5′-TAACTGAAGGCGAGCATGGG-3′
		R: 5′-CCTCTCTTCATCCGCTGGAC-3′
Superoxide dismutase-2 (SOD-2)	NM_017051.2	F: 5′-AATCAACAGACCCAAGCTAGGC-3′
		R: 5′-CACAATGTCACTCCTCTCCGAA-3′
Heme oxygenase-1 (HO-1)	XM_039097470.1	F: 5′-GTAAATGCAGTGTTGGCCCC-3′
		R: 5′-ATGTGCCAGGCATCTCCTTC-3′
Nuclear factor erythroid 2-related factor-2 (Nrf-2)	NM_031789.3	F: 5′-TTGTAGATGACCATGAGTCGC-3′
		R: 5′-TGTCCTGCTGTATGCTGCTT-3′
Protein kinase B (AKT)	XM_006240631.3	F: 5′-ACCTCTGAGACCGACACCAG-3′
		R: 5′-AGGAGAACTGGGGAAAGTGC-3′
Mammalian target of rapamycin (mTOR)	NM_019906.2	F: 5′-GACAACAGCCAGGGCCGCAT-3′
		R: 5′-ACGCTGCCTTTCTCGACGGC-3′
Inducible nitric oxide synthase (iNOS)	XM_039085203.1	F: 5′-ATGGAACAGTATAAGGCAAACACC-3′
		R: 5′-GTTTCTGGTCGATGTCATGAGCAAAGG-3′
Signal transducer and activator of transcription-3 (STAT-3)	XM_006247257.4	F: 5′-GGGCCTGGTGTGAACTACTC-3′
		R: 5′-ATGGTATTGCTGCAGGTCGT-3′
Beta actin (β-actin)	NM_031144.3	F: 5′-CCGCGAGTACAACCTTCTTG-3′
		R: 5′-CAGTTGGTGACAATGCCGTG-3′

## Data Availability

The datasets generated and/or analyzed in the present study are included in the manuscript.
